# Evaluation of mineral content in healthy permanent human enamel by Raman spectroscopy

**DOI:** 10.4317/jced.53057

**Published:** 2016-12-01

**Authors:** Anna Akkus, Asya Akkus, Renato Roperto, Ozan Akkus, Thiago Porto, Sorin Teich, Lisa Lang

**Affiliations:** 1 PhD, Department of Comprehensive Care, School of Dental Medicine, Case Western Reserve University, Cleveland, OH 44106, USA; 2 Case Biomanufacturing and Microfabrication Laboratory, Case Western Reserve University, Cleveland, OH 44106, USA; 3 DDS, MSc, PhD, Department of Comprehensive Care, School of Dental Medicine, Case Western Reserve University, Cleveland, OH 44106, USA; 4 PhD, Department of Mechanical and Aerospace Engineering, Case Western Reserve University, Cleveland, OH 44106, USA; 5 DDS, MSc, PhD, Department of Restorative Dentistry, Faculty of Dentistry, Sao Paulo State University, Araraquara, SP, Brazil; 6 DDS, MBA, Department of Comprehensive Care, School of Dental Medicine, Case Western Reserve University, Cleveland, OH 44106, USA

## Abstract

**Background:**

An understanding of tooth enamel mineral content using a clinically viable method is essential since variations in mineralization may serve as an early precursor of a dental health issues, and may predict progression and architecture of decay in addition to assessing the success and effectiveness of the remineralization strategies.

**Material and Methods:**

Twenty two human incisor teeth were obtained in compliance with the NIH guidelines and site specifically imaged with Raman microscope. The front portion of the teeth was divided into apical, medium and cervical regions and subsequently imaged with Raman microscope in these three locations.

**Results:**

Measured mineralization levels have varied substantially depending on the regions. It was also observed that, the cervical enamel is the least mineralization as a populational average.

**Conclusions:**

Enamel mineralization is affected by a many factors such as are poor oral hygiene, alcohol consumption and high intake of dietary carbohydrates, however the net effect manifests as overall mineral content of the enamel. Thus an early identification of the individual with overall low mineral content of the enamel may be a valuable screening tool in determining a group with much higher than average caries risk, allowing intervention before development of caries. Clinically applicable non-invasive techniques that can quantify mineral content, such as Raman analysis, would help answer whether or not mineralization is associated with caries risk.

** Key words:**Enamel, Raman spectroscopy, mineral content, dental caries.

## Introduction

Enamel is the hardest and the stiffest tissue in the human body. It withstands chewing forces and protects the internal dentin and pulp. It is loaded in a complex fashion throughout the life cycle of an individual. Enamel mineralization is an important property that positively correlates with the mechanical behavior of other tissues such as bone (1) and teeth (2,3). Dental enamel is 95% mineral and 1% organic matter and 4-5% water by weight percentage (4). A reduction in mineral content has direct consequences as far as dental ailments are concerned. For example, molar-incisor hypo mineralization increases tooth sensitivity to food, drinks, and thermal changes as well as results in restoration failure (5). Since variation in mineralization may manifest through white lesion formation, high or lower levels of mineralization may impact the tooth esthetics. Moreover, previous studies suggest a possible relationship between enamel mineral concentration and caries susceptibility (6,7).

Several authors have investigated enamel mineral content (8-12) using various characterization methods in the context of decay (6,7), and demineralization/ remineralization processes (13,14), age (15,16) and disease (17,18). However, the methods cited above are limited to *ex vivo* laboratory conditions and they are destructive to the specimens.

Raman spectroscopy offers the opportunity to study enamel mineral content *in vivo* (19,20), non-destructively and site-specifically. While Raman spectroscopy has been applied in the literature to assess tooth mineralization, there are no studies in the literature that examine mineralization of the enamel systematically and provide an understanding of baseline variations that may be inherent in the healthy enamel from individual to individual. The current study employed Raman spectroscopy *ex-vivo* to identify the variations enamel mineral content between individuals and site to site variations within a given tooth.

## Material and Methods

-Sample Preparation

**Figure 1:**
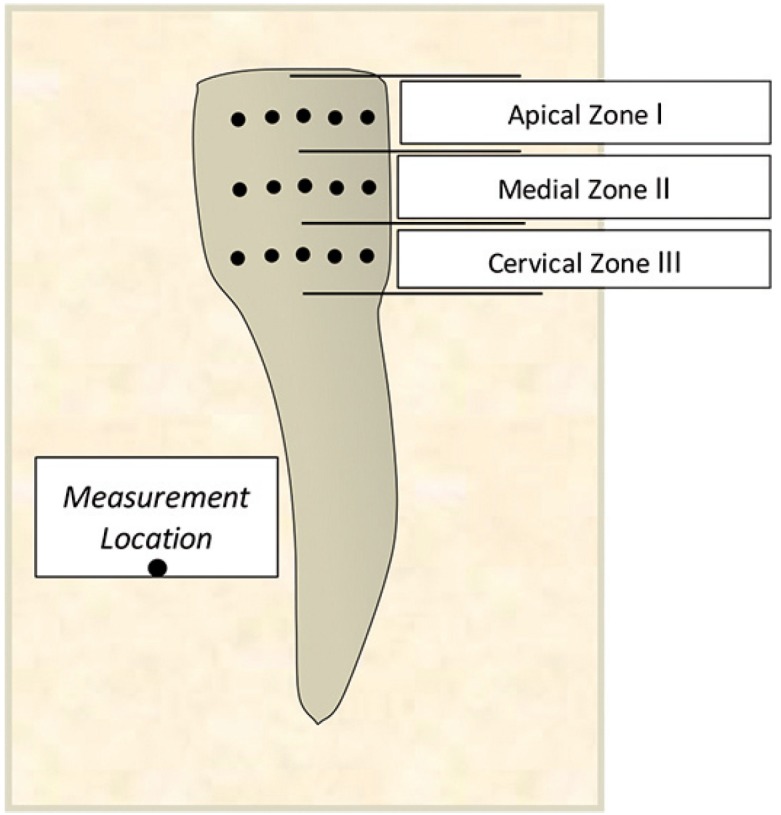
Schematic of the regions included in the Raman analysis.

Human teeth were obtained in compliance with the National Institute of Health guidelines. The Institutional Review Board exemption was filed and approved (Protocol#: EM-13-17). Eleven adult human incisors were extracted as a part of a normal treatment plan. The teeth were collected fresh within the date of the extraction and kept moist at all times without any additional disinfecting treatment. A dentist assessed the enamel of the specimens selected for Raman analysis in order to ensure that healthy intact enamel was evaluated. The samples were wrapped in wet tissue paper individually and stored in a -20 ºC freezer. Prior to Raman analysis the specimens were thawed at room temperature for 30 minutes while being wrapped in moist tissue paper. Consequently the specimens were securely positioned horizontally with resin putty in a plastic petri dish that was lined with aluminum foil to prevent stray Raman signal emerging from the plastic. A wet tissue paper was wrapped around the specimen to prevent dehydration during Raman scan, while exposing the region of interest for Raman analysis. Each tooth was measured with a ruler and the length of the crown was divided into 3 zones along the y axis: apical, medial and cervical as shown in figure 1. Three measurements were taken within each zone in order to obtain the average mineralization within each zone.

-Raman Spectroscopy

In Raman analysis, laser light is focused on a sample using a lens or objective. The reflected photons carry information on the type and the amount of chemical bonds. In the case of mineralized tissues, the phosphate group in the mineral of the enamel scatters Raman signals very strongly. Therefore, the amplitude of the phosphate peak in the Raman spectrum is proportional to the amount of mineral content. That is why, Raman spectroscopy has long been a powerful tool for assessing mineral content not only in the dentistry, but also other mineralized tissues such as bone. In the current study, an x10 objective was used to focus the laser light. The resulting excitation spot was about 10 µm in diameter and the penetration of the laser was within 100 µm.

A Raman microscope (Labram Xplora, Horiba Jobin Yvon, Edison, NJ) with laser source at 785 nm was employed. Measurements were performed using a 1200 lines/mm grating which provided a wavenumber resolution of 1.25 pixels/cm-1. Six acquisitions per point were taken. The Raman wavenumber shift measured by the system was calibrated using the known 520.7 cm-1 peak of a Si wafer. The mineralization was assessed based on the intensity of the 960 cm-1 peak of the phosphate (21,22) symmetric stretch band (23).

-Statistical Analysis

The general linear model, a multivariate analysis of variance test, was performed (Minitab, College Park, PA) to investigate the effects of position within a tooth on mineral content. When the zone-to-zone and tooth-to-tooth variations were significant Tukey’s post-hoc test was performed to check the significance of the differences between zones or individuals. The data distribution was normal as confirmed by Anderson-Darling normality test.

## Results

**Figure 2:**
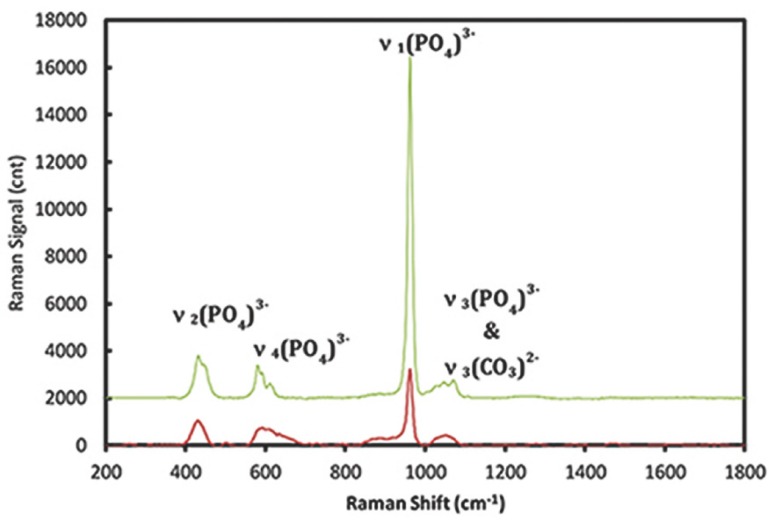
Raman spectra of incisors with the highest and lowest mineralization scores. The spectrum was shifted vertically for the sake of clarity.

**Figure 3:**
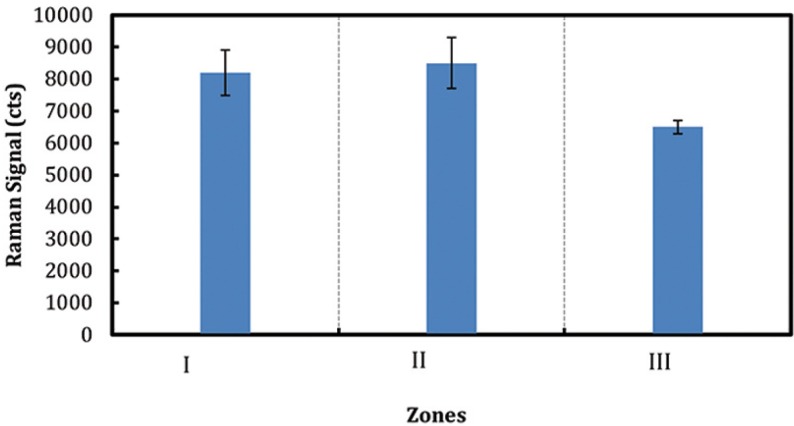
Average incisor mineralization for each zone after pooling the data for twenty two individuals.

The height of the 960 cm-1 phosphate peak was measured to compare the mineral content levels in 22 incisors. The highest Raman-based mineralization intensity was about 5-fold greater than the lowest measured mineral content, (Fig. 2). All the measurements for corresponding zone from 22 individuals with average number of measurements n=5 per tooth specimen were pooled to generate average populational value of enamel mineral content. Incisor mineral content varied dramatically depending on the regions,when the measurements were pooled within a zone, mineralization level of Zones I and II (apical, medial parts of the crown, respectively) did not differ significantly, (Fig. 3). However Zone III, the cervical region, exhibited lower mineralization (*p* < 0.05) than Zones I and II.

## Discussion

The results demonstrated that mineralization varies substantially between individuals. It was also observed that, the cervical enamel is the least mineralization as a populational average. The lower mineralization scores for the cervical region may be associated with hygiene index. Dental plaque can be normally found on the cervical area of the tooth. Due to a high acidic nature, dental plaque can easily deactivate the enamel buffering capacity. This is a process when the enamel releases calcium to normalize the saliva pH again, decreasing its mineral content. This creates spaces in enamel which are more opaque and/or whiter. Thus, it is important to understand the differences in degrees of mineralization of different tooth areas when comparing the apical, the middle and cervical areas of the outer enamel. However, the studies in the literature (8-12) are focused on investigating the enamel region in cross-section that required tooth dissection. Raman based monitoring of the mineralization in cervical region may help detect emerging issues and mitigating them.

A range of values of mineral concentration has been reported (24-30) in the literature (EDS, XRD analysis, X-ray microtomography). However, the methods employed require destruction of the specimens, such as sectioning of the tooth in slices. Therefore they are not applicable clinically. While computed tomography is viable, the spatial resolution of clinically available cone-beam systems is insufficient to capture the enamel layer. Subjection of the patient to ionizing radiation is another limitation. The Raman spectroscopy based assessment of mineralization holds a substantial promise for clinical application since Raman spectroscopy is noninvasive and sample preparation free. It is particularly informative in evaluation of the mineral content of enamel since the structural contribution of non-mineral components in mature enamel is small. In the clinical setting, Raman spectroscopy is applied by fiber optic probes to discern the enamel organization employing Raman in the polarized mode. Similar Raman probe analysis can be used to measure mineralization scores.

It has been suggested that the lower mineral concentration may be translated into increased porosity and is possibly linked to higher caries susceptibility (6,7). In addition, some studies (7,8) hypothesized that mineral concentration may be a factor determining rate of demineralization/remineralization as well. Epidemiological studies demonstrate that children from lower social background have higher caries rate (7). It is unknown whether enamel mineralization also plays a role in the greater caries-risk in this population. Other effectors of enamel mineralization are poor oral hygiene, alcohol consumption and high intake of dietary carbohydrates. An early identification of the individual with overall low mineralization of the enamel may be a valuable screening tool in determining a group with much higher than average caries risk, allowing intervention before development of caries.

## Conclusions

Non-invasive, sample preparation free Raman spectroscopy was successfully employed to measure mineral content of healthy enamel. It was demonstrated that cervical region of the healthy enamel in the pool of the incisor specimens exhibit the lowest mineralization content in the cervical region of the crown. The overall level of enamel mineral content may serve as a robust predictor of patients’ susceptibility to developing caries, thus granting the opportunity to prevent the caries via clinically available methods of remineralization, fluoride treatment as well as frequent cleaning.
